# CK2 activity is crucial for proper glucagon expression

**DOI:** 10.1007/s00125-024-06128-1

**Published:** 2024-03-20

**Authors:** Emmanuel Ampofo, Mandy Pack, Selina Wrublewsky, Anne S. Boewe, Aliya F. Spigelman, Hanna Koch, Patrick E. MacDonald, Matthias W. Laschke, Mathias Montenarh, Claudia Götz

**Affiliations:** 1 Institute for Clinical and Experimental Surgery, Saarland University, Homburg, Germany; 2 Medical Biochemistry and Molecular Biology, Saarland University, Homburg, Germany; 3 Department of Pharmacology and Alberta Diabetes Institute, University of Alberta, Edmonton, AB Canada

**Keywords:** Glucagon, Glucose homeostasis, Pancreatic alpha cells, PDX1, Protein kinase CK2

## Abstract

**Aims/hypothesis:**

Protein kinase CK2 acts as a negative regulator of insulin expression in pancreatic beta cells. This action is mainly mediated by phosphorylation of the transcription factor pancreatic and duodenal homeobox protein 1 (PDX1). In pancreatic alpha cells, PDX1 acts in a reciprocal fashion on glucagon (GCG) expression. Therefore, we hypothesised that CK2 might positively regulate GCG expression in pancreatic alpha cells.

**Methods:**

We suppressed CK2 kinase activity in αTC1 cells by two pharmacological inhibitors and by the CRISPR/Cas9 technique. Subsequently, we analysed GCG expression and secretion by real-time quantitative RT-PCR, western blot, luciferase assay, ELISA and DNA pull-down assays. We additionally studied paracrine effects on GCG secretion in pseudoislets, isolated murine islets and human islets. In vivo, we examined the effect of CK2 inhibition on blood glucose levels by systemic and alpha cell-specific CK2 inhibition.

**Results:**

We found that CK2 downregulation reduces GCG secretion in the murine alpha cell line αTC1 (e.g. from 1094±124 ng/l to 459±110 ng/l) by the use of the CK2-inhibitor SGC-CK2-1. This was due to a marked decrease in *Gcg* gene expression through alteration of the binding of paired box protein 6 (PAX6) and transcription factor MafB to the *Gcg* promoter. The analysis of the underlying mechanisms revealed that both transcription factors are displaced by PDX1. Ex vivo experiments in isolated murine islets and pseudoislets further demonstrated that CK2-mediated reduction in GCG secretion was only slightly affected by the higher insulin secretion after CK2 inhibition. The kidney capsule transplantation model showed the significance of CK2 for GCG expression and secretion in vivo. Finally, CK2 downregulation also reduced the GCG secretion in islets isolated from humans.

**Conclusions/interpretation:**

These novel findings not only indicate an important function of protein kinase CK2 for proper GCG expression but also demonstrate that CK2 may be a promising target for the development of novel glucose-lowering drugs.

**Graphical Abstract:**

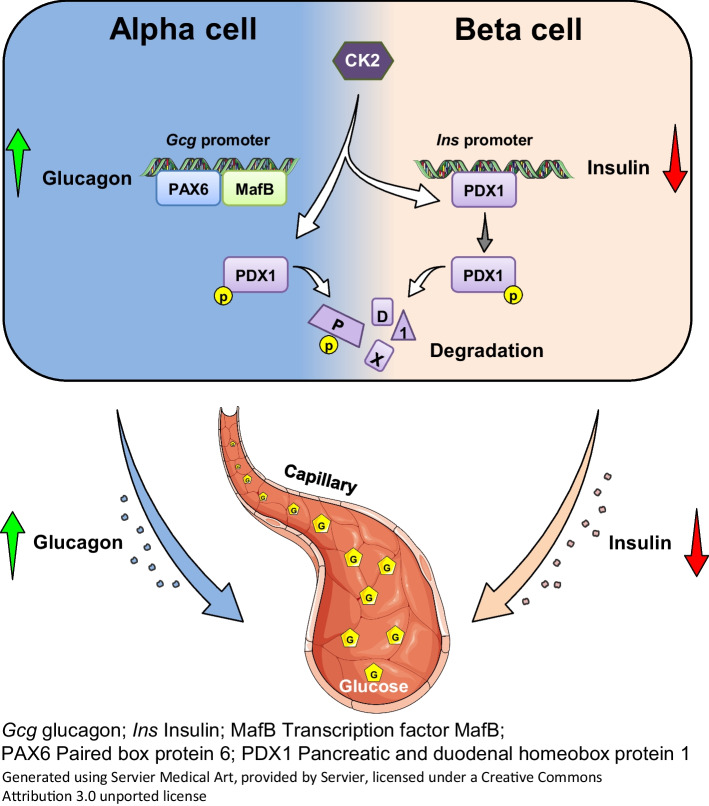

**Supplementary Information:**

The online version contains peer-reviewed but unedited supplementary material available at 10.1007/s00125-024-06128-1.


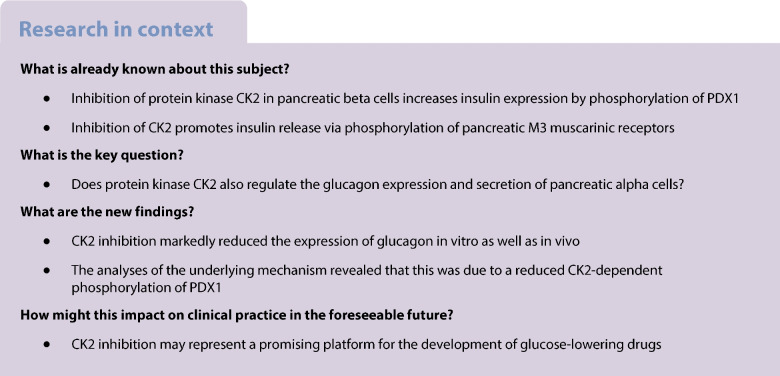


## Introduction

The pancreatic islet hormones insulin and glucagon (GCG) act antagonistically on the regulation of the blood glucose level. Insulin mainly stimulates glucose clearance by binding to its corresponding receptor on peripheral tissues [1]. In contrast, GCG increases blood glucose levels by stimulation of hepatic glycogenolysis and gluconeogenesis [2]. Consequently, blood glucose is maintained by an interplay between the hypoglycaemic action of insulin and the hyperglycaemic action of GCG.

In past decades, intensive efforts have been made to study glucose metabolism under physiological and pathological conditions with the main focus on the regulatory mechanisms of insulin expression [3–5]. The transcriptional control of insulin gene expression is well characterised [6]. Several elements in the enhancer and promoter region, as well as the associated key transcription factors, have been identified [6, 7]. One of these key players is the pancreatic and duodenal homeobox protein 1 (PDX1). This transcription factor is not only crucial for the maturation and development of pancreatic beta cells but also a vital regulator of embryonic pancreas development [3, 8]. Protein kinase 2 (CK2) is a ubiquitously expressed serine/threonine kinase, consisting of two catalytic CK2α or CK2α′ and two regulatory CK2β subunits [9], that phosphorylates the transcription factor PDX1 at threonine residue T231 and serine residue S232 [10, 11]. This results in a decreased nuclear localisation and diminished transcriptional activity of PDX1 [10, 11]. In line with this finding, Rossi et al [12] demonstrated that CK2 phosphorylates the muscarinic M3 receptor (M3R) in beta cells and that the absence of this phosphorylation ameliorates M3R-stimulated insulin release. These observations clearly indicate the important role of CK2 in insulin-mediated regulation of blood glucose levels [13]. Whether CK2 also modulates the expression of other islet hormones, such as GCG, is still unclear.

The expression and processing of pro-GCG in alpha cells result in different endocrine active peptides [2, 14], with GCG as the major bioactive product [14]. Although the *GCG* promoter was identified more than three decades ago [15], cellular mechanisms controlling pro-GCG expression in alpha cells still remain elusive. The minimal *GCG* promoter and the enhancer region contain binding sites for various transcription factors, such as hepatocyte nuclear factor 3 (HNF3), MafB, paired box protein 6 (PAX6) and PDX1 [16–19]. However, in contrast to insulin, the ectopic expression of PDX1 suppresses GCG expression [19].

Based on our previous results showing a suppressive effect of CK2 on insulin expression in beta cells [10, 11], we hypothesise that CK2 might stimulate GCG expression in alpha cells. To test this, we pharmacologically inhibited CK2 kinase activity and knocked out CK2α in murine pancreatic alpha cells. We studied the effect of CK2 inhibition or loss on the secretion, expression and transcriptional control of GCG. Putative paracrine effects of insulin on GCG expression were examined ex vivo. Moreover, we verified the effect of CK2 inhibition on GCG expression in human islets and analysed the impact of CK2 on GCG-mediated glucose homeostasis in a mouse model.

## Methods

### Materials

Collagenase NB 4G was purchased from SERVA Electrophoresis (Heidelberg, Germany). Neutral red solution, Tween20 and Hoechst 33342 were purchased from Sigma-Aldrich (Taufkirchen, Germany). Ketamine (Ursotamin) was purchased from Serumwerke Bernburg (Bernburg, Germany) and xylazine (Rompun) from Bayer (Leverkusen, Germany). BSA and FCS were purchased from Santa Cruz Biotechnology (Heidelberg, Germany). Cell lysis reagent QIAzol and QuantiNova Reverse Transcription Kit were purchased from Qiagen (Hilden, Germany). HepatoQuick, as well as water-soluble tetrazolium (WST) and lactate dehydrogenase (LDH) assays, were purchased from Roche (Basel, Switzerland). The qScriber cDNA Synthesis Kit and ORA SEE qPCR Green ROX L Mix were purchased from HighQu (Kraichtal, Germany). The CK2 inhibitors CX-4945 and SGC-CK2-1, and linsitinib (OSI-906) were purchased from SelleckChem (Munich, Germany) and Sigma-Aldrich (Taufkirchen, Germany), respectively. Protein assay dye reagent and luminol-enhanced chemiluminescence (ECL) western blotting substrate were purchased from Bio-Rad Laboratories (Feldkirchen, Germany). Lipofectamine 3000, DMEM (1 g glucose/l [5.5 mmol/l] or 4.5 g glucose/l [25 mmol/l]) and RPMI medium 1640 (2 g glucose/l [11 mmol/l]) were purchased from Fisher Scientific (Schwerte, Germany). The μMacs Streptavidin Kit was purchased from Miltenyi Biotec (Gladbach, Germany). Biotinylated primers were obtained from Life Technologies (Darmstadt, Germany).

### Antibodies

The anti-GCG antibody (catalogue no. ab92517), the anti-pAktS129 antibody (catalogue no. ab133458) and the anti-PAX6 antibody (catalogue no. ab109233) were from Abcam (Cambridge, UK). The anti-GAPDH antibody (catalogue no. 60004) and the insulin antibody (catalogue no. 15848-1-AP) were from Proteintech Germany (St Leon-Rot, Germany). The anti-Akt antibody (11E7, catalogue no. 4685) was from Cell Signaling Technology (Frankfurt am Main, Germany). The anti-CK2β antibody (E9, catalogue no. sc-46666) was from Santa Cruz Biotechnology (Heidelberg, Germany). Fructose 1,6-bisphosphatase (FBP1) was detected with a monoclonal rabbit antibody (D2T7F, catalogue no. 59172, Cell Signaling Technology). The anti-MafB (catalogue no. A700-046) antibody was from Bethyl Laboratories (Montgomery, TX, USA). Equal loading was verified using a mouse monoclonal α-tubulin antibody (clone DM1A, catalogue no. T9026, Sigma-Aldrich, München, Germany).The generation of the anti-CK2α antibody has been described previously [20]. PDX1 was identified with a polyclonal antiserum generated by immunising rabbits with recombinant mouse PDX1 [11]. The rabbit polyclonal serum no. 36 against nucleolin was generated by immunising rabbits with a C-terminal peptide of nucleolin (amino acids 696–707) [21]. The peroxidase-labelled anti-rabbit antibody (NIF 824) and the peroxidase-labelled anti-mouse antibody (NIF 825) were from GE Healthcare (Freiburg, Germany).

### Cell culture

The murine pancreatic alpha cell line αTC1 clone 6 (ATCC: CRL-2934) was cultivated in DMEM (1 g/l glucose) supplemented with 10% (vol./vol.) FCS in a humidified atmosphere with 5% CO_2_ at 37°C. The murine beta cell line MIN6 [22] was maintained in DMEM (4.5 g/l glucose) supplemented with 10% (vol./vol.) FBS in a humidified atmosphere with 5% CO_2_ at 37°C. To study the effect of CK2 on pro-GCG expression, we knocked out the catalytic CK2α subunit in αTC1 cells (αTC1 KO) by the CRISPR/Cas9 technique using the plasmid pD1431-Apuro:441627 with a guide RNA for mouse CK2α designed by ATUM (Newark, USA). Transfection was carried out using Lipofectamine 3000 (Fisher Scientific) according to the manufacturer’s instructions. After 48 h, cells were exposed to puromycin (2 µg/ml), which was renewed every 3 days, to create a stable cell line (αTC1 KO cells). In addition, the αTC1 cells were exposed to CX-4945 (10 µmol/l), SGC-CK2-1 (10 µmol/l) or DMSO as control for 24 h. Transient transfection was done with the GCG promoter construct or different p3xFlagCMV7.1-PDX1-constructs [23] and Lipofectamine 3000 according to the manufacturer’s protocol. Used cell lines were free from mycoplasma contamination.

### Western blot analysis

For the generation of whole cell extracts, αTC1 and αTC1 KO cells, as well as αTC1 cells exposed to CX-4945, SGC-CK2-1 or DMSO as control for 24 h, were harvested and lysed for 30 min at 4°C with lysis buffer (10 mmol/l Tris-HCl, pH 7.5, 10 mmol/l NaCl, 0.1 mmol/l EDTA, 0.5% [vol./vol.] Triton X-100, 0.02% [wt/vol.] NaN_3_) supplemented with 0.5 mmol/l phenylmethylsulfonyl fluoride (PMSF) and a protease and phosphatase inhibitor cocktail (1:75 vol./vol., Sigma-Aldrich). The cytoplasmic and nuclear extracts were generated and analysed as described previously in detail [24]. After electrophoresis and blotting onto PVDF membranes by a semi-dry blot procedure using a BioRad Trans-Blot-Turbo transfer system (BioRad, Munich Germany), the membranes were blocked using TBS (20 mmol/l Tris-HCl, pH 7.5, 150 mmol/l NaCl) supplemented with 0.1% (vol./vol.) Tween 20 (TBS-T) and 5% BSA (wt/vol.) for 1 h at room temperature. For the detection of proteins, primary antibodies were diluted 1:1000 with TBS-T and 1% BSA (wt/vol.) and incubated for 1 h at room temperature or overnight at 4°C. Subsequently, membranes were washed twice with TBS-T, and then incubated with the horseradish peroxidase-conjugated secondary antibodies (anti-rabbit antibody [NIF 824] or anti-mouse antibody [NIF 825]) at a dilution of 1:10,000 in TBS-T 1% BSA (wt/vol.) for 1 h at room temperature. After two further washing steps, the expression of the corresponding proteins was visualised by enhanced chemoluminescence using the ECL western blotting substrate from Bio-Rad Laboratories.

### Real-time quantitative RT-PCR

For real-time quantitative RT-PCR (qRT-PCR), total RNA from αTC1 cells as well as isolated murine islets exposed to CX-4945, SGC-CK2-1 or DMSO for 24 h were extracted using QIAzol lysis reagent. The corresponding cDNA was synthesised from the total RNA by QuantiNova Reverse Transcription Kit and the qRT-PCR analysis was performed by means of ORA SEE qPCR Green ROX L Mix (highQu, Kraichtal, Germany). Primer sequences for qRT-PCR were coded as follows: *Ins1* forward 5′-AACAACTGGAGCTGGGAGGAAG-3′ and reverse 5′-GGTGCAGCACTGATCCACAATG-3′; *Gapdh* forward 5′-CGGTGCTGAGTATGTC-3′ and reverse 5′-TTTGGCTCCACCCTTC-3′; and *Gcg* forward 5′-TGGACTCCCGCCGTGCTCAAG-3′ and reverse 5′-CCTTTGCTGCCTGGCCCTCC-3′.

### Growth curves

αTC1 and αTC1 KO cells were seeded in a 24-well plate at a density of 1×10^5^/well. After 24 h, 48 h and 72 h, cells were detached, stained with Trypan Blue solution (0.4%, wt/vol.) and counted by a LUNA automated Cell Counter (Logos Biosystems, Villeneuve D’Ascq, France) according to the manufacturer’s protocol.

### WST-1 assay

A WST-1 assay was used to analyse the effect of CK2 inhibition/loss on αTC1 and αTC1 KO cell viability by determining the activity of mitochondrial dehydrogenases. Cells were seeded in a 96-well culture plate at a density of 2×10^3^/well. After 24 h, 10 μl of WST-1 reagent (Roche) was added into each well and the absorbance was measured at 450 nm in a Tecan Infinite 200 Pro microplate reader (Tecan, Crailsheim, Germany).

### LDH assay

An LDH assay was used to evaluate the cytotoxic effects of CK2 downregulation. αTC1 and αTC1 KO cells were seeded in a 96-well culture plate at a density of 2×10^3^/well. After 24 h, an LDH assay was performed according to the manufacturer’s protocol (Roche). LDH-reaction mix (100 μl per 100 μl medium) was added to each well. After 10 min incubation, 50 μl stop solution was added and absorbance was measured at 492 nm in a Tecan Infinite 200 Pro microplate reader (Tecan, Crailsheim, Germany).

### Reporter luciferase assay

The sequence of the mouse *Gcg* promoter was amplified using murine genomic DNA (primers: forward 5′-GTACCTGAGCTCGCTAGCCGACCCTCAAATGAGACTAGG-3′ and reverse 5′-CAACAGTACCGGATTGCCAAGCTGCCCTTCTGCACCAGGGTG-3′). The resulting 379 bp construct was cloned into the XhoI restriction site of the luciferase reporter vector pGL4.10 (Promega, Mannheim, Germany). The identity of pGL4.10-*Gcg* was verified by sequencing. The transcriptional activity of the *Gcg* promoter was assessed by reporter gene assays according to the manufacturer’s instructions (Promega). Briefly, αTC1 or αTC1 KO cells were seeded in a 24-well plate. The culture medium (DMEM, 1 g glucose/l) was renewed on the day of transfection and the inhibitor (10 µmol/l CX-4945 or 10 µmol/l SGC-CK2-1) was added. Subsequently, cells were transfected with pGL4 or pGL4-*Gcg* reporter vector by using Lipofectamine 3000 for 24 h. In addition, pGL4-*Gcg*-transfected αTC1 cells were exposed to CX-4945, SGC-CK2-1 or DMSO for 24 h. Then, cells were lysed and the luciferase activity was detected by a luminescence plate reader.

### DNA pull-down assay

To study the transcription factor binding capacity to the *Gcg* promoter, we used the μMacs Streptavidin Kit and biotinylated primers (forward 5′-biotin-GACCCTCAAATGAGACTAGG-3′ and reverse 5′-biotin-GCCCTTCTGCACCAG-3′) for the generation of a biotinylated DNA probe ranging from −329 to +8 of the murine *Gcg* gene. For the pull-down assay, 1 mg of nuclear extracts were preincubated with salmon sperm DNA (100 µg/ml) for 20 min at 4°C. Thereafter, 1 µg of the biotinylated DNA probe was added and incubated for 30 min at 4°C. Fifty microlitres of streptavidin–agarose beads were added and incubated for 12 min at room temperature. The DNA–protein complex was passed through an equilibrated μMacs column, the column was washed and proteins were eluted. The eluates were then analysed by the western blot method.

### Pseudoislet formation

Pseudoislets (PIs) were generated by the liquid-overlay technique in 96-well plates covered with 1% agarose as described previously in detail [25]. Briefly, 3500 MIN6 cells and 1500 αTC1 or αTC1 KO cells were seeded per well and incubated for 5 days. The culture medium (DMEM 4.5 g glucose/l) was changed every second day. After 5 days of incubation at 37°C and 5% CO_2_, the spheroids were harvested and stored for immunohistochemical analyses or ELISA.

### Immunohistochemical analyses

PIs from MIN6 cells and either αTC1 or αTC1 KO cells were incubated for 45 min at 37°C in 100 µl HepatoQuick, 50 µl human citrate plasma and 10 µl 10% CaCl_2_ solution. The resulting clot was also fixed for 24 h in 4% (wt/vol.) paraformaldehyde at 4°C. After dehydration, the paraffin-embedded samples were cut into sections (3 μm thick). Antigens in samples were demasked by citrate buffer and the unspecific binding sites were blocked by goat serum. Cells were stained with specific primary antibodies (1:300), which were detected by the corresponding fluorescence-coupled secondary antibodies (1:1000). Cell nuclei were stained with Hoechst 33342. The sections were analysed using a BX60F fluorescence microscope (Olympus, Hamburg, Germany).

### Animals

All animal care and experimental procedures were performed according to the German legislation on protection of animals and the National Institutes of Health (NIH) Guide for the Care and Use of Laboratory Animals (Institute of Laboratory Animal Resources, National Research Council, Washington DC, USA). The experiments were approved by the local governmental animal protection committee (Landesamt für Verbraucherschutz LAV, Saarland, permit number 18/2017 and 46/2018). Mice were maintained on a standard 12 h light–dark cycle. Standard pellet chow (Altromin, Lage, Germany) and water were provided ad libitum. C57BL/6J mice (RRID:IMSR_JAX:000664, The Jackson laboratory, USA; https://www.jax.org/strain/000664) with an age of 3–12 months were used as donors for islet isolation, as recipients for transplantation and for treatment with the CK2 inhibitor CX-4945. Details of the mice used are found in electronic supplementary material (ESM) Table 1.

### Isolation of pancreatic islets

Murine pancreatic islets were isolated by collagenase-induced enzymatic digestion and purified by hand picking as described previously in detail [25]. Isolated islets were cultivated in RPMI 1640 supplemented with 10% (vol./vol.) FCS, 100 U/ml penicillin and 0.1 mg/ml streptomycin for 24 h at 37°C and 5% CO_2_. For the determination of insulin secretion, we used ten islets per well of a 24 well plate and for the determination of GCG secretion 20 islets per well of a 24 well plate.

Human islets were isolated at the Alberta Diabetes Institute IsletCore [26] and incubated overnight in DMEM supplemented with l-glutamine, 110 mg/l sodium pyruvate, 10% (vol./vol.) FCS and 100 U/ml penicillin/streptomycin (15140, Gibco) for 24 h at 37°C and 5% CO_2_. All human islet studies were approved by the Human Research Ethics Board (Pro00013094; Pro00001754) at the University of Alberta and all families of organ donors provided written informed consent. Details about the donors are shown in the human islet checklist (ESM).

### Kidney capsule model

The kidney capsule model was performed as previously described in detail [27]. Sham-transplanted mice, which were subjected to the operation procedure but did not receive any transplants, served as control. Briefly, normoglycaemic C57BL/6J mice were anaesthetised using ketamine and xylazine and 5×10^6^ αTC1 or αTC1 KO cells were injected under the left kidney capsule. Body weights and fasting blood glucose levels (7 h fasting period) of the mice were measured twice a week during the entire observation period of 28 days. Blood samples were taken from the tail vein and analysed by a portable blood glucose monitoring system (GL50; Breuer, Ulm, Germany).

### IPGTT

An IPGTT was performed on day 28 after cell transplantation under the kidney capsule of mice [25, 28]. After 14 h of fasting, the mice were intraperitoneally injected with a 10% glucose (wt/vol.) solution (10 µl/g body weight). Tail-vein blood glucose levels were determined after 0, 15, 30, 45, 60, 120 and 180 min using a portable blood glucose monitoring system (GL50; Breuer).

### Mouse islet and cell line GCG and insulin secretion measurements

For GCG secretion, αTC1 and αTC1 KO cells, murine islets or PIs from MIN6 cells and either αTC1 or αTC1 KO cells were incubated in KRB buffer (135 mmol/l NaCl, 3.6 mmol/l KCl, 5 mmol/l NaHCO_3_, 0.5 mmol/l NaH_2_PO_4_, 0.5 mmol/l MgCl_2_, 1.5 mmol/l CaCl_2_, 10 mmol/l HEPES, pH 7.4, BSA [0.1%] [wt/vol.]) with 25 mmol/l glucose for 1 h [29]. Subsequently, the buffer was removed and the cells, islets or PIs were incubated in KRB buffer containing 0.5 mmol/l glucose for 2 h.

For insulin secretion, cells, islets or PIs were incubated in KRB buffer for 1 h. Subsequently, the buffer was removed and the cells, islets or PIs were incubated in KRB buffer containing 25 mmol/l glucose for 1 h.

The incubation buffer was collected and any residual cells, islets or PIs were removed from the incubation buffer by centrifugation at 250 *g* for 10 min at 4°C. The supernatant fractions were collected and GCG and insulin secretion were determined by specific ELISA kits according to the manufacturer’s protocol (Invitrogen by Fisher Scientific, Schwerte, Germany).

### Analysis of plasma GCG and insulin levels and FBP1 expression in mice

C57BL/6J mice were given a daily i.p. injection of CX-4945 (1.5 mg/kg dissolved in DMSO/PBS) for 3 days. Mice were killed and the blood, kidney and liver samples were collected. GCG secretion was analysed by a GCG or an insulin ELISA as described above. For extraction of protein from mouse liver and kidney, the tissue sample was frozen in liquid nitrogen and crushed in a mortar. The broken tissue was resuspended in three volumes of RIPA buffer (50 mmol/l Tris/HCl, pH 8.0, 150 mmol/l NaCl, 0.5% sodium desoxycholate [wt/vol.], 1% Triton X-100 [vol./vol.], 0.1% sodium dodecylsulphate [wt/vol.]) with the protease and phosphatase inhibitor cocktail Complete (1:25) and the phosphatase inhibitor PhosSTOP (1:10) (both from Roche Diagnostics, Mannheim, Germany). The suspension was incubated on ice for 30 min. After lysis, the debris was removed by centrifugation (30 min, 4°C, 12,500 *g*). The protein content was determined according to a modified Bradford method (Bio-Rad, Munich, Germany).

### Human islet GCG and insulin secretion measurements

Isolated human islets were perfused using a BioRep perifusion system (BioRep, Miami, FL, USA). Batches of 35 islets were preincubated in perifused KRB buffer containing 140 mmol/l NaCl, 3.6 mmol/l KCl, 2.6 mmol/l CaCl_2_, 0.5 mmol/l NaH_2_PO_4_, 0.5 mmol/l MgSO_4_, 5 mmol/l HEPES, 2 mmol/l NaHCO_3_ and 0.5 mg/ml essentially fatty acid free BSA (Sigma A6003) for 30 min at 5.5 mmol/l glucose, and then perfused in KRB with changes in glucose, 100 nmol/l glucose-dependent insulinotropic polypeptide (GIP) (Anaspec, Fremont, USA), 10 mmol/l alanine (Sigma, Oakville, ON, Canada) and KCl as indicated. Samples were collected at intervals of 120–300 s and stored at −20°C until assay of GCG (U-PLEX Mouse Glucagon Assay, no. K1525YK, Meso Scale Diagnostics, Rockville, MD, USA). The insulin concentration in the same samples was determined using the Stellux Insulin Chemiluminescence ELISA (80-INSHU-CH01, Alpco, Salem, New Hampshire, USA).

### Statistical and database analysis

All in vitro experiments were reproduced at least three times. The in vivo experiments were performed with at least six animals per group and no mice were excluded from the statistical analysis. After testing the data for normal distribution and equal variance, differences between two groups were assessed by the unpaired Student’s *t* test; one-way ANOVA was applied when comparing multiple groups. This was followed by the Tukey post hoc test by means of Prism software 8 (GraphPad, USA). The results were expressed as mean ± SD. Statistical significance indicated as *p<0.05, **p<0.01 and ***p<0.001.

We utilised the PhosphoSitePlus (www.phosphosite.org, accessed 12 January 2023) and Scansite 4.0 (https://scansite4.mit.edu, accessed 12 January 2023) tools to identify the CK2 phosphorylation sites in PAX6 and transcription factor MafB.

## Results

### Effect of CK2 downregulation on proliferation, viability and GCG expression of αTC1 cells

**Fig. 1:**
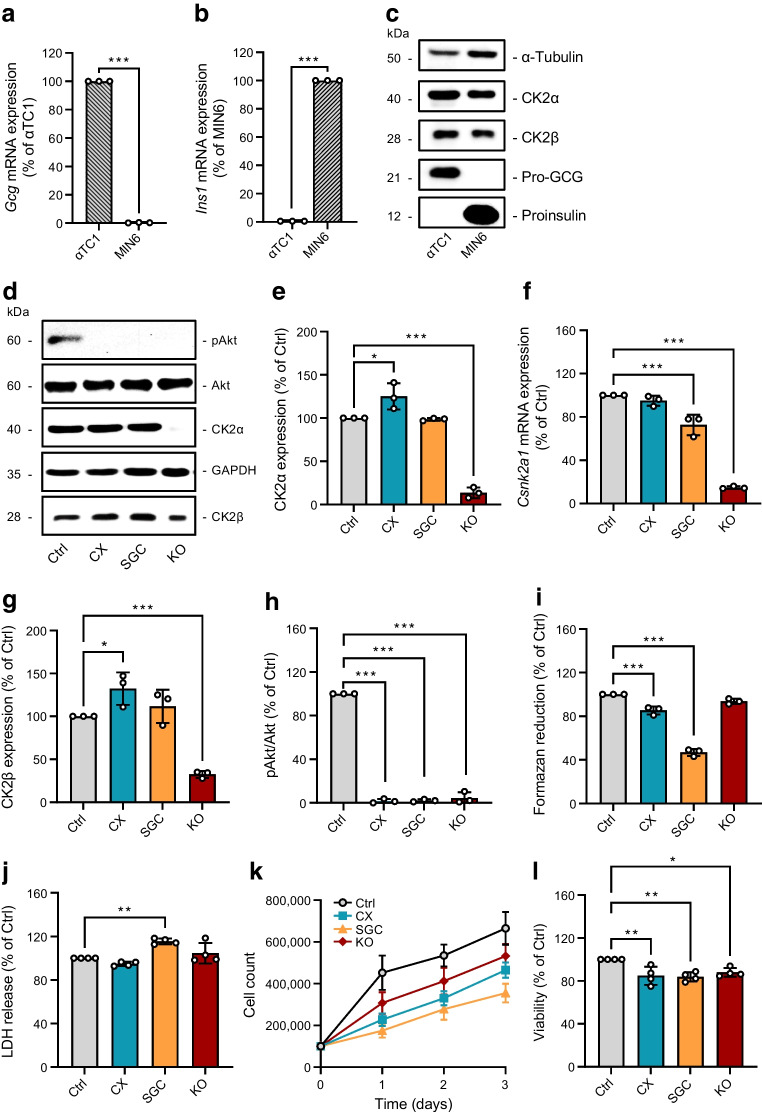
Effect of CK2 downregulation on proliferation and viability of αTC1 cells. (**a**, **b**) Quantitative analysis of *Gcg* (**a**) and *Ins1* (**b**) mRNA expression in αTC1 and MIN6 cells (*n*=3 each). (**c**) Representative western blots of α-tubulin, CK2α, CK2β, pro-GCG and proinsulin expression from whole cell extracts of αTC1 and MIN6 cells. (**d**) Representative western blots of pAkt, Akt, CK2α, GAPDH and CK2β expression from whole cell extracts of WT αTC1 cells exposed to CX-4945, SGC-CK2-1 or DMSO (control) for 24 h, as well as from αTC1 KO cells. (**e**) Quantitative analysis of CK2α expression shown in (**d**) (*n*=3 each). (**f**) Quantitative analysis of *Csnk2a1* mRNA expression in αTC1 cells as described in (**d**) (*n*=3 each). (**g**, **h**) Quantitative analysis of CK2β and pAkt/Akt expression shown in (**d**) (*n*=3 each). (**i**, **j**) αTC1 cells were treated as described in (**d**) and the viability was analysed by a WST-1 assay (**i**) and LDH assay (**j**) (*n*=3 each). (**k**, **l**) αTC1 cells were treated as described in (**d**), the cell number was determined after 1, 2 and 3 days (**k**) and the cell viability, measured by Trypan Blue exclusion assay, was assessed on day 3 (**l**) (*n*=3 each). Data are shown as mean ± SD. *p<0.05, **p<0.01, ***p<0.001. Ctrl, control (DMSO); CX, CX-4945; KO, αTC1 KO cells; SGC, SGC-CK2-1

To check whether the murine pancreatic alpha cell line αTC1 is an appropriate model to study the impact of CK2 on GCG expression and secretion, we first analysed the expression of GCG and CK2 in αTC1 cells. These cells express high levels of pro-GCG when compared with the insulin-expressing beta cell line MIN6, which served as negative control (Fig. 1a–c). Moreover, we detected both CK2 subunits, the catalytic CK2α and the regulatory CK2β (Fig. 1c). Accordingly, the αTC1 cell line was deemed suitable to study the effect of CK2 inhibition on GCG expression. The pharmacological inhibitors CX-4945 [30] and SGC-CK2-1 [31] were used to specifically repress CK2 kinase activity. In addition, we knocked out *Csnk2a1* in αTC1 cells by means of the CRISPR/Cas9 technique (αTC1 KO cells). We found that the two inhibitors had only minor effects on CK2 protein expression, whereas the deletion of the catalytic subunit abolished CK2α expression and markedly reduced CK2β protein levels (Fig. 1d–g). We further determined the effect of CK2 inhibition or loss on its catalytic activity by analysing phosphorylation of Akt on serine 129, a specific CK2 phosphorylation site [32]. As expected, we did not detect Akt phosphorylation in αTC1 cells exposed to CX-4945 and SGC-CK2-1 or in αTC1 KO cells when compared with control DMSO-treated cells (Fig. 1d,h).

**Fig. 2:**
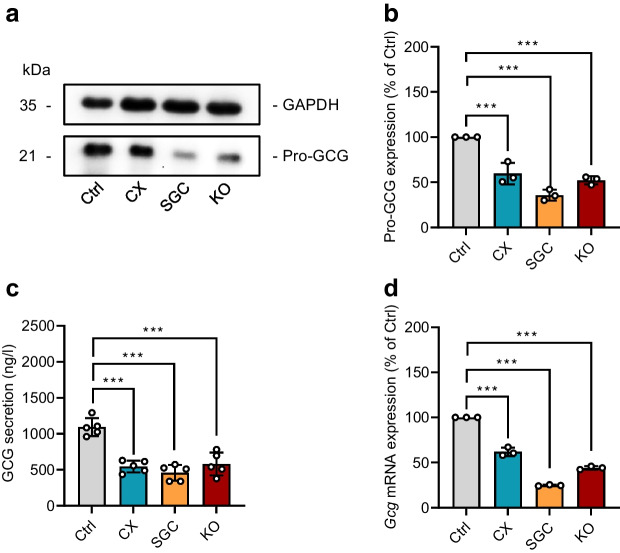
Effect of CK2 downregulation on GCG expression and secretion in αTC1 cells. (**a**) Representative western blots of pro-GCG and GAPDH expression from whole cell extracts of WT αTC1 cells exposed to CX-4945, SGC-CK2-1 or DMSO (control) for 24 h, as well as αTC1 KO cells. (**b**) Quantitative analysis of pro-GCG expression shown in (**a**) (*n*=3 each). (**c**) Quantitative analysis of GCG secretion (ng/l) from αTC1 cells treated as described in (**a**) (*n*=5 each). (**d**) Quantitative analysis of *Gcg* mRNA expression in αTC1 cells treated as described in (**a**) (*n*=3 each). Data are shown as mean ± SD. ***p<0.001. Ctrl, control (DMSO); CX, CX-4945; KO, αTC1 KO cells; SGC, SGC-CK2-1

Next, we assessed the effect of CK2 on alpha cell viability and proliferation by a panel of different assays. Our results showed that CX-4945 and SGC-CK2-1 decrease the activity of mitochondrial dehydrogenases and that this activity was not affected by the loss of CK2α (Fig. 1i). Furthermore, inhibition or loss of CK2 did not increase LDH release in αTC1 cells, except for when SGC-CK2-1 was used for inhibition (Fig. 1j). The analyses of growth curves and Trypan Blue dye exclusion revealed that both pharmacological inhibition and loss of CK2α slightly reduced αTC1 cell proliferation and viability (Fig. 1k,l). It has already been shown that CK2 inhibition promotes insulin expression [33]. Therefore, we assumed that CK2 inhibition may suppress pro-GCG expression. Indeed, the exposure of αTC1 cells to CX-4945 and SGC-CK2-1, as well as the loss of CK2α in αTC1 cells, markedly decreased pro-GCG expression (Fig. 2a,b). This, in turn, resulted in a markedly reduced GCG secretion (Fig. 2c). Whereas the median secretion of untreated cells was 1094±124 ng/l, it was reduced to 545± 82 ng/l by the use of CX-4945, 459±110 ng/l by the use of SGC-CK2-1, and 582± 160 ng/l in αTC1 KO cells. Additional analyses demonstrated that the inhibition or loss of CK2 reduced *Gcg* mRNA levels (Fig. 2d). This indicates that CK2 most probably regulates *Gcg* expression at the transcriptional level.

### Effect of CK2 downregulation on ***Gcg*** gene expression in αTC1 cells

**Fig. 3:**
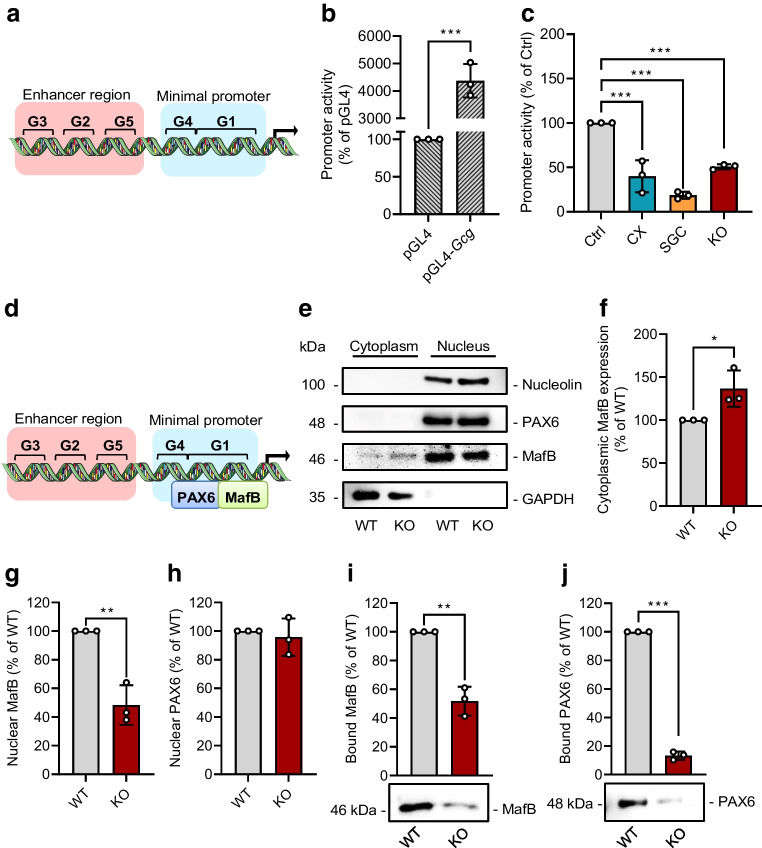
Effect of CK2 downregulation on *Gcg* gene expression in αTC1 cells. (**a**) Schematic illustration of the enhancer region (G3, G2 and G5) and the minimal promoter (G4 and G1) of the *Gcg* gene. (**b**) αTC1 cells were transfected with pGL4-*Gcg* or pGL4 empty vector as control for 24 h, cells were lysed and the promoter activity was detected by a luciferase assay (*n*=3 each). (**c**) αTC1 cells were transfected with pGL4-*Gcg* for 24 h and subsequently exposed to CX-4945, SGC-CK2-1 or DMSO (control) for 24 h; αTC1 KO cells were transfected with pGL4-*Gcg* for 24 h (*n*=3 each). The cells were lysed and the activity was detected by a luciferase assay. (**d**) Schematic illustration of the binding of PAX6 and MafB to the G1 element of the *Gcg* gene. (**e**) Representative western blots of nucleolin, PAX6, MafB and GAPDH expression from cytoplasmic and nuclear extracts of WT αTC1 cells and αTC1 KO cells. (**f**–**h**) Quantitative analysis of cytoplasmic MafB (**f**), nuclear MafB (**g**) and nuclear PAX6 (**h**) expression from cells shown in (**e**) (*n*=3 each). (**i**, **j**) Representative western blots of MafB (**i**) and PAX6 (**j**) from DNA pull-down assay, together with quantitative analysis (*n*=3 each). Data are shown as mean ± SD. *p<0.05, **p<0.01, ***p<0.001. (**a**, **d**) Generated using Servier Medical Art, provided by Servier, licensed under a Creative Commons Attribution 3.0 unported license. Ctrl, control (DMSO); CX, CX-4945; KO, αTC1 KO cells; SGC, SGC-CK2-1

To gain further insight into the mechanisms underlying CK2-regulated GCG expression, we generated a luciferase reporter construct containing the enhancer region (boxes G3, G2 and G5 in Fig. 3a) and the minimal promoter (boxes G4 and G1 in Fig. 3a) of the murine *Gcg* promoter. This construct was highly active in αTC1 cells (Fig. 3b) and its activity was dramatically reduced after inhibition or downregulation of CK2 (Fig. 3c). The G1 box of the promoter is crucial for alpha cell specificity and *Gcg* gene expression [34]. It has been reported that the transcription factors PAX6 and MafB bind to this box, resulting in an enhanced *Gcg* transcription (Fig. 3d) [35]. Hence, we analysed the nuclear localisation of both transcription factors, depending on CK2 activity. We detected an increased cytoplasmic and decreased nuclear localisation of MafB in αTC1 KO cells when compared with αTC1 wild-type (WT) cells (Fig. 3e–g). In contrast, the nuclear localisation of PAX6 was not affected by CK2 downregulation (Fig. 3e,h). We next performed a DNA pull-down assay to study the binding capacity of the two transcription factors in WT αTC1 cells and αTC1 KO cells. As expected, MafB and PAX6 bound to the promoter in WT αTC1 cells; however, this binding was markedly reduced after CK2α loss (Fig. 3i,j).

### Effect of CK2 downregulation on PDX1-mediated ***Gcg*** gene expression in αTC1 cells

**Fig. 4:**
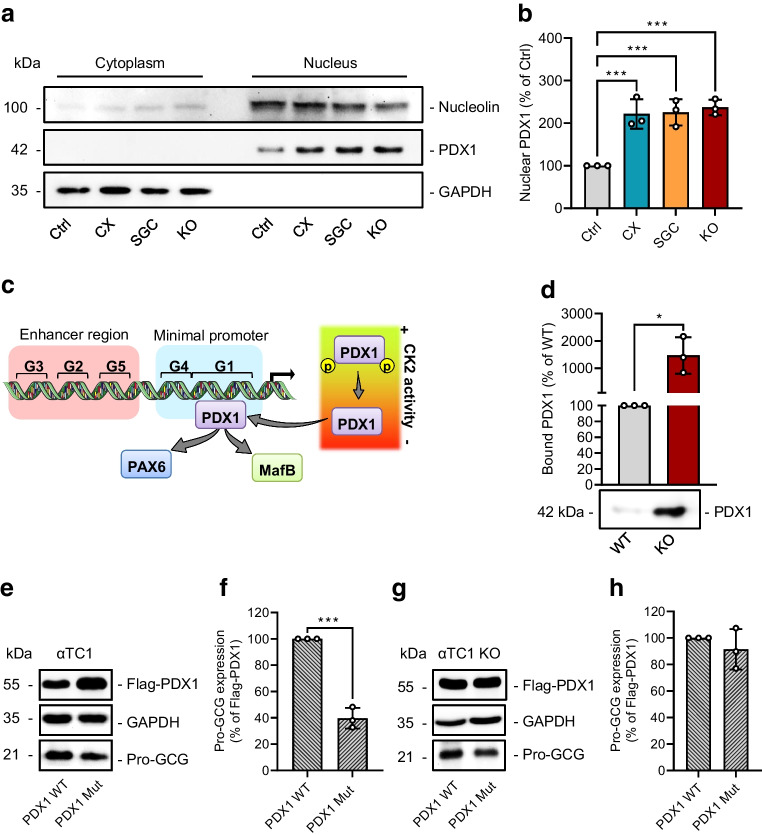
Effect of CK2 downregulation on PDX1-mediated *Gcg* gene expression in αTC1 cells. (**a**) Representative western blots of nucleolin, PDX1 and GAPDH expression from cytoplasmic and nuclear extracts of WT αTC1 cells exposed to CX-4945, SGC-CK2-1 or DMSO (control) for 24 h, as well as αTC1 KO cells. (**b**) Quantitative analysis of nuclear PDX1 expression from cells shown in (**a**) (*n*=3 each). (**c**) Schematic illustration of the orchestrated dissociation of PAX6 and MafB and CK2-mediated binding of PDX1 to the G1 element of the *Gcg* gene. (**d**) Representative western blot of PDX1 from a DNA pull-down assay and quantitative analysis of PDX1 from the indicated western blot (*n*=3 each). (**e**) Representative western blots of Flag-PDX1, GAPDH and pro-GCG expression from whole cell extracts of αTC1 cells overexpressing PDX1 WT or PDX1 Mut. (**f**) Quantitative analysis of pro-GCG expression from cells shown in (**e**) (*n*=3 each). (**g**). Representative western blots of Flag-PDX1, GAPDH and pro-GCG expression from whole cell extracts of αTC1 KO cells overexpressing PDX1 WT or PDX1 Mut. (**h**) Quantitative analysis of pro-GCG from cells shown in (**g**) (*n*=3 each). Data are shown as mean ± SD. *p<0.05,***p<0.001. (**c**) Generated using Servier Medical Art, provided by Servier, licensed under a Creative Commons Attribution 3.0 unported license. Ctrl, control (DMSO); CX, CX-4945; INS, insulin; KO, αTC1 KO cells; SGC, SGC-CK2-1

We have already shown that CK2 phosphorylates murine PDX1 at residues T231 and S232 and that the loss of the CK2-dependent phosphorylation stabilises and increases the transcriptional activity of PDX1 in beta cells [10, 11, 23]. Pancreatic alpha cells exhibit very low levels of PDX1 and we herein found that downregulation of CK2 increases the nuclear fraction of PDX1 (Fig. 4a,b). It has been shown that PDX1 is capable of suppressing *Gcg* gene expression in beta cells by binding to the G1 box and, thus, displacing MafB and PAX6 [34]. To address the possibility that CK2 reduces *Gcg* expression via PDX1 in αTC1 cells (Fig. 4c), we performed a DNA pull-down assay. The loss of CK2α in αTC1 KO cells significantly increased the binding of PDX1 to the *Gcg* promoter (Fig. 4d). To further analyse the importance of the CK2-specific phosphorylation of PDX1 for pro-GCG expression, we overexpressed PDX1 WT or PDX1 T231A/S232A (Mut) in αTC1 cells and determined GCG expression. In fact, we detected lower promoter activity and protein expression of pro-GCG in PDX1 Mut-overexpressing cells (Fig. 4e,f and ESM Fig. 1). On the other hand, overexpression of PDX1 WT in αTC1 KO cells did not result in higher PDX1 levels when compared with PDX1 Mut-overexpressing αTC1 KO cells (Fig. 4g,h). Taken together, these results indicate that the loss of CK2 activity reduces GCG expression in a PDX1-dependent manner.

### Effect of CK2 downregulation on insulin and GCG secretion ex vivo

**Fig. 5:**
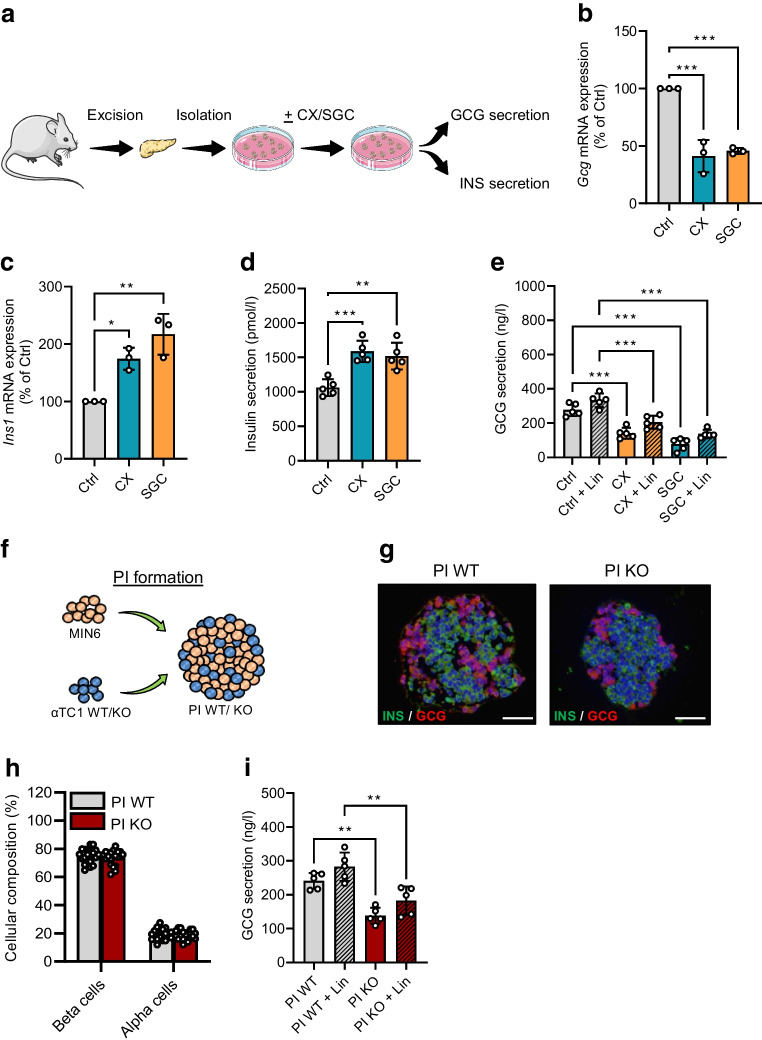
Effect of CK2 downregulation on insulin and GCG secretion ex vivo. Schematic illustration of the murine islet isolation procedure, exposure to the inhibitors and the analyses of endocrine function. (**b**, **c**) Quantitative analysis of *Gcg* (**b**) and *Ins1* (**c**) mRNA expression in isolated murine islets exposed to CX-4945, SGC-CK2-1 or DMSO (control) for 24 h (*n*=3 each). (**d**) Quantitative analysis of insulin secretion (pmol/l) from isolated murine islets (10 islets per well of 24 well plate) treated as described in (**b**) (*n*=5 each). (**e**) Quantitative analysis of GCG secretion (ng/l) from isolated murine islets (20 islets per well of a 24 well plate) exposed to CX-4945, CX-4945 + linsitinib, SGC-CK2-1, SGC + linsitinib, DMSO (control) or DMSO + linsitinib for 24 h (*n*=5 each). (**f**) Schematic illustration of PI formation from MIN6 cells and αTC1 cells (PI WT) or MIN6 cells and αTC1 KO cells (PI KO). (**g**) Representative immunofluorescence staining of insulin (green) and GCG (red) in PI WT and PI KO. Cell nuclei were stained with Hoechst 33342 (blue). Scale bar, 75 µm. (**h**) Quantitative analysis of insulin- (beta cells) and GCG- (alpha cells) positive cells (expressed as % of all PI cells) in PI WT and PI KO (*n*=20 each). (**i**) Quantitative analysis of GCG secretion (ng/l) from PI WT, PI WT exposed to linsitinib, PI KO and PI KO exposed to linsitinib (*n*=5 each). Data are shown as mean ± SD. *p<0.05, **p<0.01, ***p<0.001. (**a**, **f**) Generated using Servier Medical Art, provided by Servier, licensed under a Creative Commons Attribution 3.0 unported license. Ctrl, control (DMSO); CX, CX-4945; KO, αTC1 KO cells; INS, insulin; Lin, linsitinib; SGC, SGC-CK2-1

Next, we examined whether CK2 inhibition also decreases GCG secretion ex vivo. For this, islets were isolated from C57BL/6J mice and exposed to CX-4945 or SGC-CK2-1 to determine the expression and secretion of insulin and GCG (Fig. 5a). In line with our in vitro findings, we found a markedly reduced expression of *Gcg* after CK2 inhibition (Fig. 5b). It is known that insulin affects GCG secretion in a paracrine manner [36]. Therefore, CK2-induced insulin secretion in islets may attenuate the secretory capacity of alpha cells. In line with our previous studies [33], we here demonstrated that the pharmacological inhibition of CK2 promoted *Ins1* expression as well as insulin secretion (Fig. 5c,d). However, results from isolated islets exposed to either of the two inhibitors, with or without the insulin receptor blocker linsitinib, clearly showed that most of the decreased GCG secretion is due to CK2 inhibition in alpha cells and only a minor effect is due to the paracrine action of insulin (Fig. 5e). We additionally strengthened our findings by the generation of PI build from MIN6 (3500 cells) and αTC1 cells (1500 cells) (PI WT) or build from MIN6 (3500 cells) and αTC1 KO cells (1500 cells) (PI KO) (Fig. 5f,g). CK2 knockout did not affect the cellular composition of the PIs (Fig. 5h). As expected, in line with the results from treatment with inhibitors, PI KO exhibited a significantly lower GCG secretion when compared with PI WT (Fig. 5i).

### Effects of CK2 downregulation in mice

**Fig. 6:**
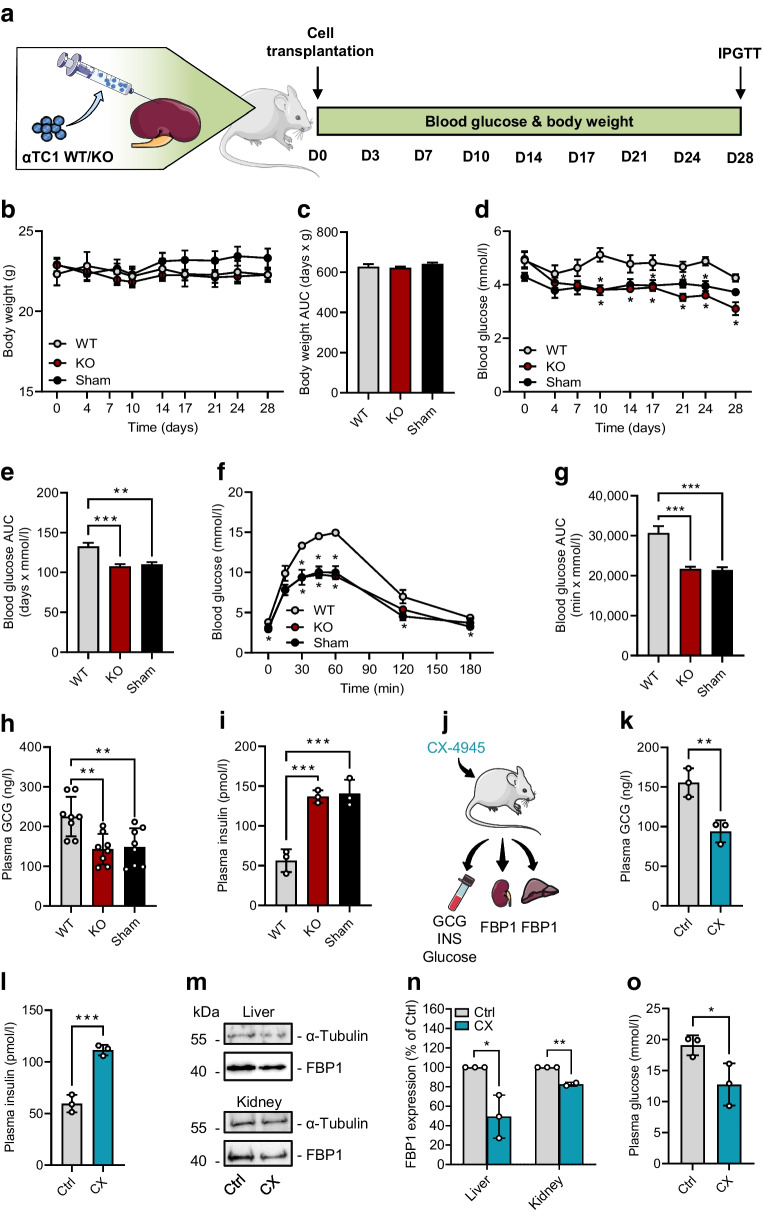
Effect of CK2 downregulation on blood glucose, hormone secretion and FBP1 expression in mice. (**a**) Schematic illustration of the experimental setting. On day 0, αTC1 cells (WT or KO) were transplanted under the left kidney capsule of mice. Sham-transplanted mice served as negative control. Fasting blood glucose levels and body weights were measured over 28 days twice a week. On day 28, IPGTT was performed. (**b**) Body weight of mice transplanted with WT or KO cells from day 0 to day 28 (*n*=6 each). Sham-transplanted mice served as negative control (*n*=5 each). (**c**) AUC of the body weights shown in (**b**). (**d**) Blood glucose levels of mice transplanted with WT or KO cells from day 0 to day 28 (*n*=6 each). Sham-transplanted mice served as negative control (*n*=5 each). (**e**) AUC of the blood glucose levels shown in (**d**). (**f**) Quantitative analysis of blood glucose levels on day 28 according to the IPGTT of mice transplanted with WT or KO cells (*n*=6 each). Sham-transplanted mice served as negative control (*n*=5 each). (**g**) AUC of IPGTT results shown in (**f**). (**h**) Quantitative analysis of GCG secretion of mice transplanted with WT or KO cells (*n*=8 each). Sham-transplanted mice served as negative control (*n*=8 each). (**i**) Quantitative analysis of insulin secretion of mice transplanted with WT or KO cells (*n*=3 each). Sham-transplanted mice served as negative control (*n*=3 each). (**j**) Schematic illustration of the experimental setting. Mice were treated for 3 days with CX-4945 or DMSO (control) and blood, kidney and liver samples were collected to study GCG and insulin secretion and FBP1 expression. (**k**) Quantitative analysis of GCG secretion of mice treated with CX-4945 or DMSO (control) (*n*=3 each). (**l**) Quantitative analysis of insulin secretion of mice treated with CX-4945 or DMSO (control) (*n*=3 each). (**m**) Representative western blots of FBP1 and α-tubulin expression from liver and kidney tissue extracts. (**n**, **o**) Quantitative analysis of FBP1 from data shown in (**m**) (*n*=2 or 3 each). Data are shown as mean ± SEM. *p<0.05, **p<0.01, ***p<0.001. (**a**, **j**) Generated using Servier Medical Art, provided by Servier, licensed under a Creative Commons Attribution 3.0 unported license. Ctrl, control (DMSO); CX, CX-4945; INS, insulin

To assess the influence of CK2 on GCG expression in vivo, we transplanted αTC1 WT or KO cells under the kidney capsule of C57BL/6J mice. Moreover, we used sham-transplanted mice as an additional control group. Fasting blood glucose levels and body weights were measured over 28 days twice a week (Fig. 6a). We did not observe any differences in the body weights between the groups (Fig. 6b,c). We measured significantly higher blood glucose levels after fasting in mice receiving αTC1 WT cells when compared with sham-transplanted mice or mice transplanted with αTC1 KO cells over the entire observation period (Fig. 6d). Accordingly, the AUC for glucose in the WT group was significantly increased when compared with that in the KO and sham groups (Fig. 6e). Based on the fasting-induced hyperglycaemia observed in the mice transplanted with αTC1 WT cells, we evaluated the effect of CK2-mediated altered GCG expression on glucose tolerance and clearance. The results of the IPGTT demonstrated higher blood glucose levels in mice receiving αTC1 WT cells (Fig. 6f,g). This is not surprising given the fact that these mice received additional alpha cells that are not under paracrine control of pancreatic islet cells, and, thus, gluconeogenesis is enhanced. Indeed, we measured elevated plasma GCG levels and lower insulin levels in mice receiving WT αTC1 cells when compared with mice receiving KO cells and the sham-transplanted group (Fig. 6h,i).

To study the effect of the reduced GCG expression after CK2 inhibition under physiological conditions, mice were treated with CX-4945 or DMSO (as control) for 3 days and plasma GCG and insulin levels were examined. As a key enzyme of gluconeogenesis, FBP1 expression in liver and kidney was analysed (Fig. 6j). As expected, we measured markedly lower plasma GCG levels and higher plasma insulin levels after CK2 inhibition (Fig. 6k,l). Moreover, we detected a diminished expression of FBP1 in liver and kidney samples of CX-4945-treated mice (Fig. 6m,n) and reduced plasma glucose levels of CX-4945-treated mice when compared with controls (Fig. 6o).

**Fig. 7:**
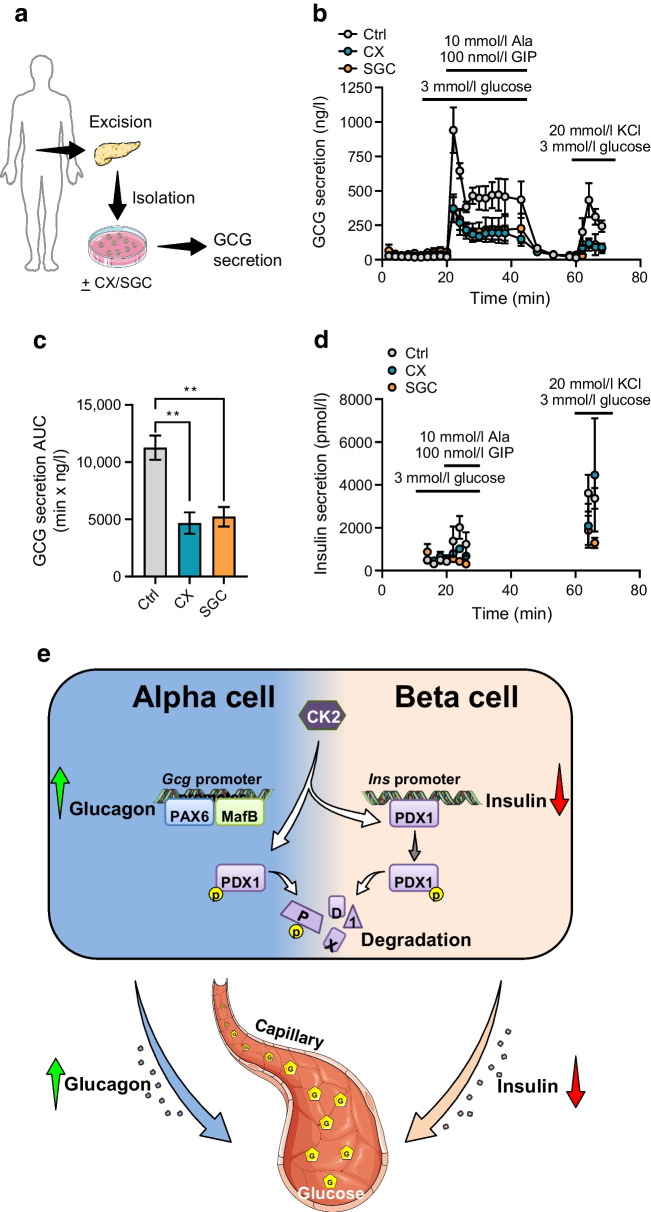
Effect of CK2 downregulation on GCG secretion from isolated human islets. (**a**) Schematic illustration of the human islet isolation procedure, exposure to the inhibitors and the analysis of the endocrine function. (**b**) Human isolated islets (*n*=3 donors) were exposed to CX-4945, SGC-CK2-1 or DMSO (control) for 24 h and GCG secretion was analysed at the indicated time points; changes in glucose, secretagogues and KCl were as indicated. (**c**) AUC of the blood glucose levels shown in (**b**). (**d**) Human isolated islets (*n*=3 donors) were exposed to CX-4945, SGC-CK2-1 or DMSO (control) for 24 h and insulin secretion was analysed at the indicated time points; changes in glucose, secretagogues and KCl were as indicated. Data are shown as mean ± SEM. **p<0.01 (**e**) The yin/yang hypothesis of the effects of CK2 on PDX1 and hormone secretion in alpha and beta cells. CK2 phosphorylates PDX1, which promotes its degradation and reduces its transcriptional activity. This results in a decreased binding of PDX1 to the insulin promoter and, thus, a decrease in insulin secretion in beta cells. In alpha cells, the CK2-dependent PDX1 phosphorylation contributes to the very low expression level of this transcription factor. This, in turn, promotes the binding of MafB and PAX6 to the *Gcg* promoter. Taken together, by this mechanism, CK2 is involved in the regulation of glucose homeostasis. (**a**, **e**) Generated using Servier Medical Art, provided by Servier, licensed under a Creative Commons Attribution 3.0 unported license. Ctrl, control (DMSO); CX, CX-4945; SGC, SGC-CK2-1

Rodent and human islets have differences in their cellular composition and intra-islet cell interactions [37]. To exclude that the observed effects of CK2 on GCG expression are specific for mice, we finally determined GCG secretion in islets from healthy human donors. For this, human isolated islets were treated with CX-4945 and SGC-CK2-1 for 24 h and subsequently GCG secretion was induced by low-glucose buffer (3 mmol/l glucose) complemented with GIP and alanine (Fig. 7a). In line with our results from αTC1 cell lines and murine islets, CK2 inhibition significantly reduced GCG secretion from human islets (Fig. 7b). Accordingly, the AUC of GCG secretion in CX-4945- and SGC-CK2-1-exposed human islets was significantly reduced compared with control islets (Fig. 7c). We additionally analysed insulin release from these islets. As expected, we detected only low levels of released insulin within all groups, and the release was not affected by 3 mmol/l of glucose (Fig. 7d). These findings indicate that CK2 also represses human *GCG* gene expression, resulting in a reduced GCG secretion.

## Discussion

A survey of our and others’ findings revealed that protein kinase CK2 acts as a negative regulator of insulin-driven glucometabolic control [11–13, 33]. Detailed analyses concerning the role of CK2 in beta cells demonstrated that this is mainly caused by a CK2-dependent phosphorylation of PDX1 and, thus, reduced stability and transcriptional activity [10]. The fact that PDX1 represses GCG expression led to our hypothesis that CK2 may promote GCG expression in pancreatic alpha cells. In the present study, we found that CK2 inhibition or loss markedly downregulates GCG expression and secretion. Investigation of the underlying mechanisms revealed an enhanced binding of PDX1 to the *Gcg* promoter. Additional in vivo experiments showed that specific loss of CK2α in pancreatic alpha cells leads to hypoglycaemia. Moreover, we could demonstrate the important role of CK2 for the endocrine function of alpha cells in isolated human islets.

Lawlor et al [38] reported in their integrative transcriptomic and epigenomic analysis that the αTC1 cell line exhibits several characteristics of pancreatic alpha cells and are suitable to study cellular endocrine functions. Therefore, we decided to use this cell line for our in vitro experiments. We showed that inhibition or loss of CK2α markedly reduces GCG expression and secretion in αTC1 cells. When investigating whether the loss or inhibition of this kinase reduces cell proliferation and viability, we did not detect any effect on cell necrosis but found a slightly decreased cell proliferation. The latter can be explained by the fact that CK2 phosphorylates several proteins involved in cell cycle control, such as CDC25B, phosphoprotein phosphatase 2A (PP2A), histone deacetylase 1/2 (HDAC1/2), and topoisomerase II α/β [39–41]. It may be conceivable that the reduced proliferation also decreases the endocrine function of αTC1 cells. However, proliferative alpha cells are not found in islets of adults [42] and our ex vivo experiments demonstrated a reduced GCG secretion after CK2 inhibition. Hence, we assume that proliferation and endocrine function of αTC1 cells can be analysed independently of each other.

In the present study, we used the pharmacological CK2 inhibitors CX-4945 [30] and SGC-CK2-1 [31]. Both inhibitors are described as CK2-specific inhibitors with a different spectrum of off-target effects [31, 43–47]. To exclude putative effects in the present study, we additionally generated a CK2α-deficient αTC1 cell line. By using these strategies, we studied the effect of CK2 activity on *Gcg* gene expression. We found that CK2 indirectly regulates its expression at the transcriptional level. The structure and transcriptional regulation of the *Gcg* promoter is well known. The *Gcg* promoter consists of five control elements (G1–G5) [34]. G1 and G4 represent the minimal promoter and DNA-binding studies exhibited that PAX6 together with MafB interact with the G1 element to trigger the alpha cell-specific expression of GCG [15, 34, 35, 48]. In the present study, we detected an attenuated binding of PAX6 and MafB to the *Gcg* promoter after CK2 inhibition. CK2 phosphorylates various transcription factors and, thus, regulates their activity [49–55]. However, the two transcription factors are not identified as CK2 substrates. In addition, in silico analyses of phosphosites-based data did not show any stringent CK2 phosphorylation sites within the polypeptide chain of PAX6 and MafB (www.phosphosite.org, https://scansite4.mit.edu, accessed 12 January 2023). Therefore, we assume that the decreased binding of the two transcription factors to the *Gcg* promoter is not due to a reduced CK2-dependent phosphorylation but rather to displacement from the promoter. It has long been known that CK2 phosphorylation near a nuclear localisation signal affects nuclear import [56]. Due to lack of a CK2 phosphorylation site in the vicinity of a nuclear localisation signal of the polypeptide chain of MafB, we suspect that inhibition of the CK2 kinase activity is not responsible for the change in subcellular localisation of MafB.

PDX1 is a homeodomain transcription factor that plays an essential role during pancreatic development and differentiation [57]. In the adult pancreas, PDX1 is highly expressed in beta cells where it promotes insulin expression [58]. In contrast, PDX1 represses GCG expression in beta cells by binding to the *Gcg* promoter element G1, thereby blocking the interaction of PAX6 and MafB with the G1 element [34]. Hence, it is not surprising that the ectopic expression of PDX1 dramatically decreases *Gcg* gene expression in pancreatic alpha cells [59] and that only low levels of PDX1 are detectable in pancreatic alpha cells [58]. In previous studies, we have demonstrated that CK2 phosphorylates PDX1 on threonine 231 and serine 232, leading to a markedly reduced transcriptional activity [10, 11, 33]. Furthermore, this phosphorylation destabilised PDX1 and promoted its degradation [23]. We herein found that inhibition or loss of CK2α not only elevates nuclear PDX1 levels but also increases PDX1 binding to the *Gcg* promoter. To exclude the possibility that these effects are mediated by other CK2 substrates, which, in turn, might affect PDX1 activity, we ectopically expressed PDX1 WT or the CK2 phosphorylation mutant PDX1 Mut (T231A/S232A) in αTC1 cells. Our results showed a significantly reduced GCG expression. This indicates that the loss of CK2 activity stabilises and enhances the transcriptional activity of PDX1, leading to displacement of PAX6 and MafB from the *Gcg* promoter. Besides PDX1, PAX6 and MafB, additional transcription factors such as hepatocyte nuclear factor 3 (HNF3), homeobox protein Nkx-6.1 (NKX6.1) and PAX4 are involved in the regulation of GCG gene expression [60]. None of these have been identified as a CK2 substrate so far.

It is known that a decreased gene transcription does not necessarily result in a decreased protein secretion. We have already demonstrated that CK2 modulates the activity of the P/Q-type calcium channel Ca_V_2.1 in beta cells [61]. This channel is not only expressed in alpha cells but also involved in GCG secretion [62]. Hence, an influence of CK2 on this voltage-gated calcium channel is also conceivable in alpha cells but this will also be dependent on the alpha cell-specific environment and will not stringently result in the same impact on activity. However, a detailed analysis of a possible interaction between CK2 and Ca_V_2.1 in alpha cells was far beyond the scope of this paper. Moreover, Bozadjieva et al [63] reported that reduced mammalian target of rapamycin complex 1 (mTORC1) signalling impairs GCG secretion, and Rajak et al described a crinophagic degradation of GCG in alpha cells [64]. It is of note that CK2 inhibition decreases mTORC1 activity [65]. In line with this, we here found an attenuated phosphorylation of mTORC1 after CK2 inhibition (ESM Fig. 2). Thus, CK2 is not a classical signal transducer in the sense that it is part of a vertical signal transduction chain. It acts in a horizontal manner and influences components of several signal transduction cascades.

Paracrine signalling in pancreatic islets adds an extra level of diversity and complexity to the endocrine physiology [66]. In islets, beta cells are in direct contact with alpha cells [60] and insulin may repress GCG secretion by direct binding to the insulin receptor on alpha cells [36, 67, 68]. Therefore, it might be possible that the insulin secretion from beta cells may decrease GCG secretion in a paracrine manner. To test this, isolated murine islets and pseudoislets were additionally exposed to linsitinib, a dual IGF-1/insulin receptor blocker. We found that only a minor reduction in GCG secretion is mediated by the paracrine effect of insulin, whereas the main effect is caused by the CK2-dependent phosphorylation of PDX1. GCG secretion stimulated by hypoglycaemia leads to glucose mobilisation through the promotion of glycogenolysis and gluconeogenesis in GCG target organs such as the liver [2]. To study the effect of CK2 inhibition on GCG-mediated regulation of blood glucose levels, we used the kidney capsule model [27]. Our results demonstrated that mice receiving αTC1 WT cell transplants display increased fasting blood glucose levels, higher CGC and lower insulin serum levels when compared with mice transplanted with no cells or αTC1 KO cells, pointing to the important role of CK2 in pancreatic alpha cells for preventing hypoglycaemia. To verify that CK2 activity regulates GCG expression in alpha cells under physiological conditions, we additionally treated mice with CX-4945 and analysed plasma GCG levels as well as the gluconeogenic activity of the liver and kidney by the expression of FBP1, the expression of which is regulated by GCG [69]. As expected, we detected lower levels of plasma GCG, higher levels of plasma insulin and a reduced expression of FBP1 in CX-4945-treated mice, when compared with controls. However, it should be noted that GCG may also stimulate insulin secretion from pancreatic beta cells, raising insulin levels [70, 71].

Although GCG and CK2α are encoded by evolutionarily highly conserved genes, different regulatory mechanisms in alpha cells between human and rodents are conceivable, because human and murine islets differ in their cellular composition as well as intra-islet cell interactions [37]. In human islets, the ratio of beta cells to alpha cells is lower than in mice [37]. Furthermore, a distinct cell distribution is lacking. In fact, alpha, beta and delta cells appear to be randomly distributed throughout the entire human islet [72]. In contrast, murine islets have a core consisting of beta cells and the other endocrine cells are localised in the mantle region [72]. Nevertheless, despite these differences, we also measured a significantly diminished GCG secretion after CK2 inhibition in human islets.

Hyperglycaemia that occurs in individuals with type 2 diabetes mellitus is caused by different abnormalities such as an impaired insulin secretion, GCG secretion, glucose uptake/utilisation/reabsorption or lipotoxicity. Hence, drugs targeting different cellular mechanisms to reduce hyperglycaemia have been developed during recent decades. Currently, there are several classes of orally administered pharmacological agents available for the therapy of type 2 diabetes, such as sulfonylureas, metformin, dipeptidyl peptidase IV (DPP-4) inhibitors and oral glucagon-like peptide 1 (GLP-1) receptor agonists [73]. These can be used as monotherapy or as a combination of two or more drugs from other classes to increase the glucose-lowering mode of action. We and other groups have already shown that CK2 inhibition reduces hyperglycaemia by increasing insulin expression and secretion [10, 12, 33]. In the present study, we found that CK2 inhibition lowers blood glucose levels by reducing GCG expression and, thus, reducing gluconeogenesis. These findings not only demonstrate the important role of CK2 in the regulation of blood glucose levels by insulin and GCG (Fig. 7e) but also suggest that CK2 inhibition may represent a promising platform for the development of new glucose-lowering drugs.

### Supplementary Information

Below is the link to the electronic supplementary material.

Supplementary file1 (PDF 265 KB)

## Data Availability

Data sets are available upon reasonable request from the corresponding author.

## Abbreviations

CK2: Protein kinase CK2, formerly known as casein kinase 2
ECL: Enhanced chemiluminescence
FBP1: Fructose 1,6-bisphosphatase
GCG: Glucagon
GIP: Glucose-dependent insulinotropic polypeptide
HNF3: Hepatocyte nuclear factor 3
LDH: Lactate dehydrogenase
M3R: M3 receptor
PAX6: Paired box protein 6
PDX1: Pancreatic and duodenal homeobox protein 1
PDX1 Mut: PDX1 T231A/S232A mutation
PI: Pseudoislet
qRT-PCR: Real-time quantitative RT-PCR
TBS-T: TBS supplemented with Tween 20
WST: Water-soluble tetrazolium
WT: Wild-type
