# Supplementary material for: CK2 activity is crucial for proper glucagon expression
Source: Diabetologia. 2024 Mar 20;67(7):1368–85. doi: 10.1007/s00125-024-06128-1 (PMC11153270; doi:10.1007/s00125-024-06128-1)
Supplement: Supplementary file 1 — Supplementary file1 (PDF 265 KB) [file 125_2024_6128_MOESM1_ESM.pdf]

ESM Table 1: Details about the used C57BL/6J mice

Experiment	Number	Sex	Age (weeks)	Body weight (g)
**Donor: qRT-PCR**	1	female	42	27
	1	female	48	29
	1	male	40	31
	1	male	37	31
**Donor: secretion**	2	female	44	25-29
	1	female	20	20
	1	female	29	24
	1	male	40	33
	1	male	13	21
	1	male	28	29
**Recipient: DMSO**	1	female	16	19
	2	male	12	20-22
**Recipient: CX-4945**	1	female	20	21
	2	male	13	21-22
**Recipient: αTC1 WT**	3	female	24	20-23
	3	male	17	22-26
**Recipient: αTC1 CK2α KO**	3	female	24	21-23
	3	male	19	23-25
**Recipient: sham**	3	female	24	20-24
	2	male	20	22-26

ESM Figure 1

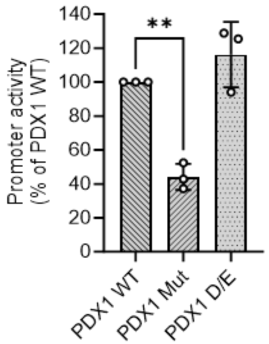

ESM Fig. 1. αTC1 cells were transfected with pGL4-*Gcg*, as well as p3xFlag-CMV7.1.-PDX1 wildtype (PDX1 WT), p3xFlag-CMV7.1.- PDX1 T231A/S232A (PDX1 Mut) or p3xFlag-CMV7.1.- PDX1 T231D/S232E (PDX1 D/E) for 24 h. The cells were lysed and the promoter activity was detected by a luciferase assay. Data are expressed in % of PDX1 WT (n = 3 each). Mean ± SD. **P < 0.01 vs. PDX1 WT.

ESM Figure 2

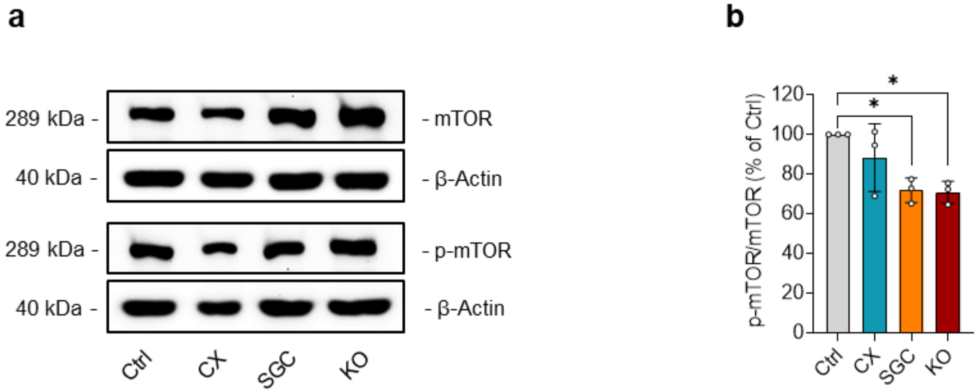

ESM Fig. 2. (**a**) Representative Western blots of mTOR, p-mTOR (phospho-Ser2448), and β-actin expression from whole cell extracts of αTC1 cells exposed to CX-4945 (CX), SGC-CK2-1(SGC) or DMSO (Ctrl) for 24 h as well as αTC1 KO cells. mTOR was detected with mTOR (7C10) rabbit monoclonal antibody (cat.no. #2983), p-mTOR with phospho-mTOR (Ser2448) (D9C2) rabbit monoclonal antibody (cat.no. #5536), both from Cell Signaling Technology, Frankfurt a. M., Germany. β-Actin served as loading control and was detected with a monoclonal mouse antibody (cat. no. hrp-66009 from Proteintech, St Leon-Rot, Germany). (**b**) Quantitative analysis of p-mTOR/mTOR expression from (a) after normalization to equal loading. Data are expressed in % of Ctrl (n = 3 each). Mean ± SD. $^*P < 0.05$ vs. Ctrl.

Checklist for reporting human islet preparations used in research

Adapted from Hart NJ, Powers AC (2018) Progress, challenges, and suggestions for using human islets to understand islet biology and human diabetes. Diabetologia https://doi.org/10.1007/s00125-018-4772-2

**Islet preparation**	**1**	**2**	**3**	**4**	**5**	**6**	**7**	**8**[a]
**MANDATORY INFORMATION**								
Unique identifier	R448	R450	R456					
Donor age (years)	61	65	59					
Donor sex (M/F)	F	F	F					
Donor BMI (kg/m^2)	36.1	21.9	28.8					
Donor HbA_{1c} or other measure of blood glucose control	5.8	4.3	5					
Origin/source of islets[b]	Alberta IsletCore	Alberta IsletCore	Alberta IsletCore					
Islet isolation centre	Alberta IsletCore	Alberta IsletCore	Alberta IsletCore					
Donor history of diabetes? Please select yes/no from drop down list	No	No	No					
**If Yes, complete the next two lines if this information is available**								
Diabetes duration (years)								
Glucose-lowering therapy at time of death[c]								
**RECOMMENDED INFORMATION**								
Donor cause of death	DCD	NDD	NDD					
Warm ischaemia time (h)								

Cold ischaemia time (h)	13.5	7.5	13					
Estimated purity (%)	95	90	80					
Estimated viability (%)								
Total culture time (h)[d]	40	30	64					
Glucose-stimulated insulin secretion or other functional measurement[e]	GSIS (1->10 mM glucose) Stimulation index 6.14	GSIS (1->10 mM glucose) Stimulation index 15.81	GSIS (1->10 mM glucose) Stimulation index 6.62					
Handpicked to purity? Please select yes/no from drop down list	Yes	Yes	Yes					
Additional notes								

[a]If you have used more than eight islet preparations, please complete additional forms as necessary

[b]For example, IIDP, ECIT, Alberta IsletCore

[c]Please specify the therapy/therapies

[d]Time of islet culture at the isolation centre, during shipment and at the receiving laboratory

[e]Please specify the test and the results
